# Histone Methyltransferase *MLL1* Mediates Oxidative Stress and Apoptosis upon Deoxynivalenol Exposure in the Intestinal Porcine Epithelial Cells

**DOI:** 10.3390/antiox11102006

**Published:** 2022-10-11

**Authors:** Dongfeng Shi, Yiyi Shan, Xiaoyang Zhu, Haifei Wang, Shenglong Wu, Zhengchang Wu, Wenbin Bao

**Affiliations:** 1 Key Laboratory for Animal Genetics, Breeding, Reproduction and Molecular Design of Jiangsu Province, College of Animal Science and Technology, Yangzhou University, Yangzhou 225009, China; 2 Joint International Research Laboratory of Agriculture & Agri-Product Safety, Yangzhou University, Yangzhou 225009, China

**Keywords:** deoxynivalenol, IPEC-J2 cells, *MLL1*, H3K4me3, transcriptome

## Abstract

Deoxynivalenol (DON), as a secondary metabolite of fungi, is continually detected in livestock feed and has a high risk to animals and humans. Moreover, pigs are very sensitive to DON. Recently, the role of histone modification has drawn people’s attention; however, few studies have elucidated how histone modification participates in the cytotoxicity or genotoxicity induced by mycotoxins. In this study, we used intestinal porcine epithelial cells (IPEC-J2 cells) as a model to DON exposure in vitro. Mixed lineage leukemia 1 (*MLL1*) regulates gene expression by exerting the role of methyltransferase. Our studies demonstrated that H3K4me3 enrichment was enhanced and *MLL1* was highly upregulated upon 1 μg/mL DON exposure in IPEC-J2 cells. We found that the silencing of *MLL1* resulted in increasing the apoptosis rate, arresting the cell cycle, and activating the mitogen-activated protein kinases (MAPKs) pathway. An RNA-sequencing analysis proved that differentially expressed genes (DEGs) were enriched in the cell cycle, apoptosis, and tumor necrosis factor (TNF) signaling pathway between the knockdown of *MLL1* and negative control groups, which were associated with cytotoxicity induced by DON. In summary, these current results might provide new insight into how *MLL1* regulates cytotoxic effects induced by DON via an epigenetic mechanism.

## 1. Introduction

Deoxynivalenol (DON), also known as vomitoxin, belongs to the Trichothecene group, which is mainly *Fusarium graminearum*, *F. culmorum* and *F. crookwellense*, and has one of the highest contamination ratio and detection rates among mycotoxins [1]. DON contaminates wheat, barley, maize, and oats, etc. Moreover, DON is hard to be degraded even in the condition of high temperature and strong acid due to its stable chemical structure [2]. DON can be “modified” to produce more derivatives, such as 3-acetyl-DON, 15-acetyl-DON, deoxynivalenol-3-beta-D-glucopyranoside (D3G), de-epoxy DON (DOM-1), 3-epi-DON, and 3-keto-DON [3]. Pigs are very susceptible to DON, especially the intestine of pigs, which is the primary target of DON attacks [4]. In China, the National Standard GB 2761-2017 has stipulated that the standard limit of DON in cereals and grain products (including corn, barley, wheat, cereal, and wheat flour) should be 1000 µg/kg. In vivo, chronic DON exposure not only led to decreased feed intake and body weight in animals, but also affected their susceptibility to diseases and vaccination efficiency. For instance, a previous study suggested that low-dose DON exposure influenced the replication of porcine epidemic diarrhea virus (PEDV), increased diarrhea rates, and exacerbated the gut barrier injury [5]. Moreover, the exposure of pigs to DON for 4 weeks reduced the efficiency of vaccination against porcine reproductive and respiratory virus (PRRSV) [6]. In vitro or in vivo, acute or chronic DON both alters the expression of tight junction proteins thereby disrupting the intestinal barrier [7,8]. Mechanically, DON inhibits protein and nuclei acid synthesis by binding to the 60 S ribosome [9]. Previous studies have shown that DON caused accumulation levels of reactive oxygen species (ROS) in the mitochondria which could active the oxidative stress response and induce apoptosis and immune dysfunction [10]. 

Epigenetics aims to explain the regulation of gene expression by altering transcriptional activity and chromosomal structure [11]. Recently many studies have shown that epigenetic regulation can be involved in a variety of biological processes, including cytotoxicity induced by mycotoxin. For instance, our previous study indicated that the genome-wide methylation levels significantly changed and found 3030 differentially methylated regions (DMRs) after the IPEC-J2 cells were treated with 1 μg/mL DON for 48 h, then combined with RNA-sequencing analysis; the promotor region methylation of 29 genes was negatively correlated with gene expression [12]. DON exposure may play an important role in DNA methylation, but the underlying mechanism remains to be clarified. Histone methylation is a highly dynamic modification regulated by histone methyltransferases and demethylases in vitro and in vivo, which can result in the activation or suppression of gene expression, depending on the specific methylation site [13]. Lysine 4 (K4) methylation on histone 3 (H3) is a wide spectrum of modification and conservatively plays a crucial role in gene expression. The trimethylation of Lysine 4 on histone 3 (H3K4me3) generally marks the transcriptional activating of genes, whilst the trimethylation of histone 3 lysine 27 is usually related to silence gene expression [14].

Mixed lineage leukemia 1 (*MLL1*), also known as KMT2A, was initially uncovered in the research of cancer and the translocation and rearrangement of the *MLL1* gene sequence results in the leukemia [15]. Subsequently, other studies discovered that *MLL1* could specifically catalyze the H3K4 to undergo methylation modification [16]. *MLL1* plays an important role in stem cell proliferation and differentiation, and regulates the expression of homeobox (HOX) gene clusters which are the major regulators during early embryonic development [17]. 

In the past several years, *MLL1* was deeply investigated in the regulation of leukemogenesis, but few studies were performed regarding its own role under mycotoxins [18]. In the present study, the intestinal porcine epithelial cells (IPEC-J2 cells) were used as an in vitro model to explore the potential mechanism regulation of *MLL1* upon DON stimulation. We uncovered that H3K4me3 modification enrichment was enhanced and the levels of *MLL1* mRNA and MLL1 protein were highly expressed, upon IPEC-J2 cells exposure to 1 μg/mL DON. Silencing the *MLL1* increased the rate of apoptosis and the level of ROS, the cell cycle was also arrested, and the MAPKs pathway was activated. Combined with the RNA-seq, TNF receptor superfamily member 1A (TNFRSF1A) is probably the potential target of *MLL1* to participate in the regulation of DON.

## 2. Materials and Methods

### 2.1. Cell Culture and Treatment

The porcine small intestinal epithelial cell line (IPEC-J2), was purchased from the American Type Culture Collection (ATCC), cultured in Dulbecco’s modified Eagle medium (DMEM, Invitrogen Corporation, Carlsbad, CA, USA) with 10% (*v*/*v*) fetal bovine serum (FBS, bio-channel, Nanjing, China) and 1% penicillin-streptomycin (solarbio, Beijing, China). All cells were placed in a 37 °C incubator with 5% CO_2_. As our previous study demonstrated, IPEC-J2 cells were treated with 1 μg/mL DON (C_15_H_20_O_6_; Sigma-Aldrich, St. Louis, MO, USA) for 48 h and a concentration of dimethyl sulfoxide (DMSO) <0.1% [12].

### 2.2. Cell Viability

To determine whether the cell growth was affected by DON, the cell viability assay was conducted using a Cell Counting Kit-8 (Yeasen Biotechnology (Shanghai) Co., Ltd., Shanghai, China) according to the protocols provided from the manufacturer. Briefly, IPEC-J2 cells (4000 cells/well) were put into 96-well culture plates. After cell confluence of approximately 50%, DON was added to the medium at a final concentration of 1 μg/mL. The cells were incubated with no FBS medium containing 10% reagent for 2 h at 37 °C. The absorbance was monitored using the Tecan Infinite 200 microplate reader (Sunrise, Tecan, Switzerland) at a wavelength of 450 nm. All experiments were performed in triplicate.

### 2.3. Immunofluorescence

The IPEC-J2 cells were seeded on the glass slide in a 12-well plate and exposed with 1 μg/mL DON for 48 h or not. The cells were fixed with 4% paraformaldehyde for 30 min, then permeabilized with 0.5% Triton X-100 for 15 min and blocked with 5% BSA for 2 h at 37 °C incubator. Afterwards, anti-alpha-tubulin (ab7291, Abcam, Cambridge, UK) was incubated with cells overnight at 4 °C, followed by the cells being washed in PBST thrice and stained with secondary antibodies for 1h at 37 °C incubator. DAPI (Invitrogen) was used to counterstain the nucleus. The cells were photographed with an inverted microscope (Olympus, Japan).

### 2.4. RNA Extraction and Real Time Polymerase Chain Reaction (RT-PCR)

Total RNA was extracted from cells after treatment using RNAiso (Takara, Dalian, China) following the instructions from the manufacturer. Next, 1 μg RNA was reversely transcribed into complementary DNA (cDNA) using a cDNA synthesis kit (Vazyme Biotech Co., Ltd., Nanjing, China). Briefly, the reactions were performed in an ABI StepONEPlus Real-Time PCR System (Applied Biosystems, Foster City, CA, USA) with a 10 μL mixture system following the instructions. All the primers used are shown in Table S2. *GAPDH* and *β-ACTIN* were chosen for normalizing the other gene levels. 2^−∆∆Ct^ method was used to calculate the relative gene expression [19].

### 2.5. Analysis Cell Cycle and Apoptosis using Flow Cytometry 

Cell apoptosis was evaluated using Annexin V-fluorescein isothiocyanate (FITC)/propidium iodide (PI) kit (Solarbio, Beijing, China). IPEC-J2 cells were transfected with si-*MLL1*-NC or si-*MLL1*, si-*TNFRSF1A*-NC or si-*TNFRSF1A*, and then treated with 1 μg/mL DON for 48 h in a 6-well plate when the cell fusion reached 50%. There are several different groups including si-*MLL1*-NC + DON, si-*MLL1* + DON, si-*TNFRSF1A*-NC and si-*TNFRSF1A*. After treatments, the samples were performed as described previously [20]. Cell apoptosis and cell cycle were detected using flow cytometry (Beckman Coulter, Brea, CA, USA), and then analyzed by CytExpert 2.3 and FlowJo 7.6. Each measurement was performed three times using a total of 10,000 events per sample.

### 2.6. Western Blot

After treatments, cells were collected and lysed with radioimmunoprecipitation assay (RIPA) buffer supplemented with protease inhibitor and phosphatase inhibitor on ice. Total protein was extracted and the concentration was measured by BCA protein assay kit (Beyotime Institute of Biotechnology, Jiangsu, China). The protein samples were diluted in 5X SDS-PAGE buffer and denatured by heating at 95 °C for 10 min. Each lane was loaded equal mass sample (20 μg). Samples were separated by 12.5% sodium dodecyl sulfate polyacrylamide gel electrophoresis, then transferred to polyvinylidene difluoride (PVDF) membranes (Immobilon, Darmstadt, Germany). Next, the membranes were blocked for 2 h at room temperature with TBST buffer containing 5% skim milk, followed by being incubated overnight at 4 °C with specific primary antibodies and then incubated for 1 h at RT with secondary antibodies (goat anti-mouse IgG HRP, goat anti-rabbit IgG HRP; CWBIO, Beijing, China). Finally, the membrane was detected by ECL and the relative intensities were analyzed by ImageJ software. 

Anti-H3K4me3 (ab8580), anti-p38 (ab170099), anti-phopsho-p38 (ab178867), anti-JNK (ab1779461), anti-phospho-JNK (ab124956) were purchased from Abcam Ltd. (Cambridge, UK). Anti-MLL1 antibody (sc-374392) was obtained from Santa Cruz Ltd. (Santa Cruz, CA, USA). Anti-ERK 1/2 (4695S) and anti-phospho-ERK 1/2 (4370S) were purchased from Cell signaling Technology Ltd. (Danvers, MA, USA). Anti-BAX (ER0907), anti-caspase 3 (ER30804), and anti-cleaved-caspase 3 (ET1608-64) were purchased form Hangzhou HuaAn Biotechnology (Hangzhou, China). Anti-HSP90 (60318-1-lg) was obtained from Proteintech Ltd. (Wuhan, China).

### 2.7. Small Interfering RNA (siRNA) Transfection

Three pig *MLL1* and *TNFRSF1A* siRNAs and a negative control siRNA sequence were synthesized from GenenPharma (Suzhou, China). The siRNA *MLL1* sequences were listed in Table S1, and the siRNAs of *TNFRSF1A* sequences are listed in Table S1, of which si-*MLL1*-3 and si-*TNFRSF1A*-3 were efficient and used in the study for analysis. Cells (1 × 10^6^ cells/well) were seeded in 6- or 12-well plates, then the transfection was performed using jet PRIME (PolyPlus, France) according to the manufacturer’s instructions when the cells had reached approximately 60–70% confluence. After transfection, the medium was changed for 24 h, and cells were harvested for another 24 h. 

### 2.8. Reactive Oxygen Species Assessment

Reactive oxygen species (ROS) levels were determined with 2′,7′-dichlorofluorescein diacetate (DCFH-DA) probe using a reactive oxygen species assay kit (Solarbio, Beijing, China). After treatments, cells were washed twice in cooled-PBS, then incubated with serum-free DMEM containing DCFH-DA (10 μM) for 30 min at 37 °C incubator. Following, fluorescence signals of DCF were measured at 488 nm excitation and 525 nm emission using the confocal microscope (Leica, Heidelberg, Germany) within 1 h.

### 2.9. RNA-Sequencing and Library Construction

Total RNA of samples transfected with si-*MLL1* and si-*MLL1*-NC was isolated using RNAiso (Takara, Dalian, China) for RNA-seq library construction. The quality of RNA was inspected with a 1% agarose gel, and the RNA integrity and concentration were monitored using a Qubit RNA Assay Kit at the Qubit^®^ 2.0 Fluorometer (Life Technologies, Camarillo, CA, USA) and a NanoDrop spectrophotometer (IMPLEN, Westlake Village, CA, USA), respectively. Afterwards, the NEBNext@ UltraTM II Directional RNA Library Prep Kit (New England Biolabs (NEB), Beijing, China) was conducted to prepare the RNA-seq library of each sample following the provided guidelines, and the libraries were sequenced on Illumina HiSeq2000 platform. The quality control of sequences raw data was filtered to discard low-quality reads using fastQC, the clean reads were aligned to the pig reference genome (Ensembl, Sscrofa11.1) using Bowtie2, the read counts mapped to each gene were calculated using HTSeq program, the FPKM (fragments per kilobase of transcript sequence per million of mapped fragments) was determined using Gfold, the differentially expressed genes (DEGs) between the samples were verified with DESeq2, and the threshold *p*-values < 0.05 and |log2 (fold change)| > 0 were considered to be statistically significant [21,22,23,24]. GO (Gene Ontology) functional annotation of DEGs was conducted with GOseq, which divided biological function of DEGs into three aspects including cellular component, molecular function, and biological process. KOBAS software was employed to check the enrichment of DEGs in the KEGG (Kyoto Encyclopedia of Genes and Genomes) pathways. The GO terms and KEGG pathways analysis were performed using a correction with cutoff of 0.05. 

### 2.10. Determination of Porcine TNFRSF1A Core Promotor Region with Reporter Assay

The 2kb upstream sequence of pig *TNFRSF1A* gene was derived from UCSC database, and then the core promotor region was predicted using the online website of Berkeley Drosophila Genome Project (BDGP) and Promotor 2.0. According to the predicted loci, the sequence was cut into four different lengths (-500-(-1) bp, -750-(-1) bp, -1010-(-1) bp and -1610-(-1) bp) to amplify corresponding products. The primers are listed in Table S3. The pGL3-basic vector was digested with *XhoI* and *HindⅢ* for 3 h, and then the four amplification fragments of the promotor were ligated in vector for 15 min at 50 ℃ using a ClonExpress Ultra One Step Cloning Kit (Vazyme biotech Co., Ltd., Nanjing, China). Next, the ligations were transformed into *Trelief^TM^ 5α* Chemically competent cells (TSINGKE, Nanjing, China) and then cultured on a solid LB-medium containing ampicillin at 37 °C overnight. Monoclonal colonies were taken and replicated in the liquid LB-medium, consequently, plasmids were extracted, and sequenced by company (Songon, Shanghai, China).

HEK293T cells were co-transfected into 500 ng *Renilla* plasmid and four recombinant plasmids and an empty vector as negative control, and afterwards cultured for 48 h at 37 °C incubator. The Substrate fluorescence intensity was detected on a Tecan Infinite 200 microplate reader (Tecan, Switzerland) using a Dual Luciferase Reporter Assay Kit (Vazyme biotech Co., Ltd., Nanjing, China). The promotor activities were calculated by the ratio *Firefly* Luciferase/*Renilla* Luciferase.

### 2.11. Chromatin Immunoprecipitation (ChIP) Assay

IPEC-J2 cells treated with 1 μg/mL DON (60% density) or not were digested (1 × 10^7^) and washed twice with chilled PBS. The cell precipitation was resuspended with medium and cells and were cross-linked with 1% formaldehyde for 10 min on a shaker at room temperature, then crosslinking was ended with 50 mM glycine for 5 min [25]. The chromatin fragments of 200–700 bp were obtained through lysing cell by lysed buffer (5 mM PIPES, pH 8.0, 85 mM KCl, 1% Nonidet P-40, 1 × protease inhibitor cocktail) and sonicated by Bioruptor (Covaris) for 21 min below 7 °C. ChIP-grade antibodies (anti-tri-methyl-histone H3 (Lys 4), ab8580, Abcam) and rabbit immunoglobulin G (IgG) were incubated with 400 μL TBST containing 50 μL Protein A/G Magnetic Beads (MedChemExpress, Monmouth Junction, NJ, USA) for 4 h on a rotator at 4 °C, and then the chromatin and the beads connected antibody were co-incubated overnight with rotation at 4 °C. Next, the ChIP DNA was de-crosslinked by Protease K digested at 65 °C overnight and enriched and purified using a FastPure Gel DNA Extraction Mini Kit (Vazyme biotech Co., Ltd., Nanjing, China). At last, purification of DNA was performed and detected via qPCR using *TNFRSF1A* primers listed in Table S3.

### 2.12. Statistical Analysis

The results were analyzed using Excel and GraphPad Prism version 8 (GraphPad Software, San Diego, CA, USA). The data were represented as means ± SD, Student’s *t*-test was applied to compare significance between two groups. *p* < 0.05 was regarded as statistically significant. 

## 3. Results

### 3.1. The Toxic Impacts of DON Exposure on IPEC-J2 Cells

**Figure 1:**
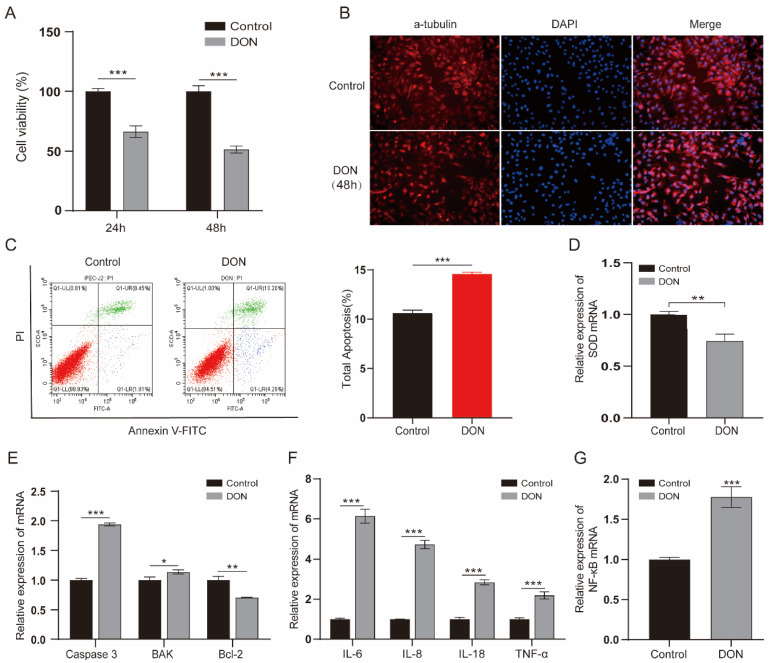
The toxic impacts of DON exposure on IPEC-J2 cells. (**A**) The viability of cells which were induced by 1 µg/mL concentration DON for 24 h and 48 h (*n* = 6 per group). (**B**) The morphological changes between treated and control group cells for 48 h 1 μg/mL DON exposure. Cells were stained by α-tubulin antibody (red) and DNA was stained by DAPI (blue). Scale bar = 100 µm. (**C**) Cell apoptosis ratio of cell samples at 48 h exposure were analyzed by flow cytometry. Annexin-FITC: green, PI: red. (**D**–**G**) RT-qPCR analyzed the mRNA levels of *SOD*, apoptosis regulators (*Caspase 3*, *BAK* and *Bcl-2*), cytokines (*IL-6*, *IL-8*, *IL-18* and *TNF-α*) and *NF-κB*. Data are shown as the mean ± SD of three independent experiments (*n* = 3); * *p* < 0.05, ** *p* < 0.01, *** *p* < 0.001 (Student’s *t*-test).

To examine the effect of DON on IPEC-J2 cells, we treated cells with a concentration of 1 µg/mL for 24 h and 48 h. The results indicated that 1 µg/mL DON significantly reduced the cell viability; specifically, the cell viability declined to about 50% at 48 h (*p* < 0.001) (Figure 1A). We further monitored the cell morphological changes of IPEC-J2 cells exposed to 1 μg/mL DON; the cells exhibited an irregular and shrinkage morphology (Figure 1B). Furthermore, apoptosis analysis found that the DON-treated group obviously increased the ratio of apoptosis compared with the control group (*p* < 0.001) (Figure 1C). qPCR assay revealed that the expression of antioxidative gene *SOD* was decreased (Figure 1D), the pro-apoptotic factors (*caspase 3* and *BAK*) were elevated and antiapoptotic factor (*Bcl-2*) was reduced (Figure 1E). We also revealed that the expression of cytokines (*IL-6*, *IL-8*, *IL-18* and *TNF-α*) and *NF-κB* were increased upon 1 μg/mL DON exposure (*p* < 0.001) (Figure 1F, G). These results suggest that DON causes toxicity in IPEC-J2 cells.

### 3.2. Elevation of H3K4me3 and MLL1 Expression Induced by DON 

**Figure 2:**
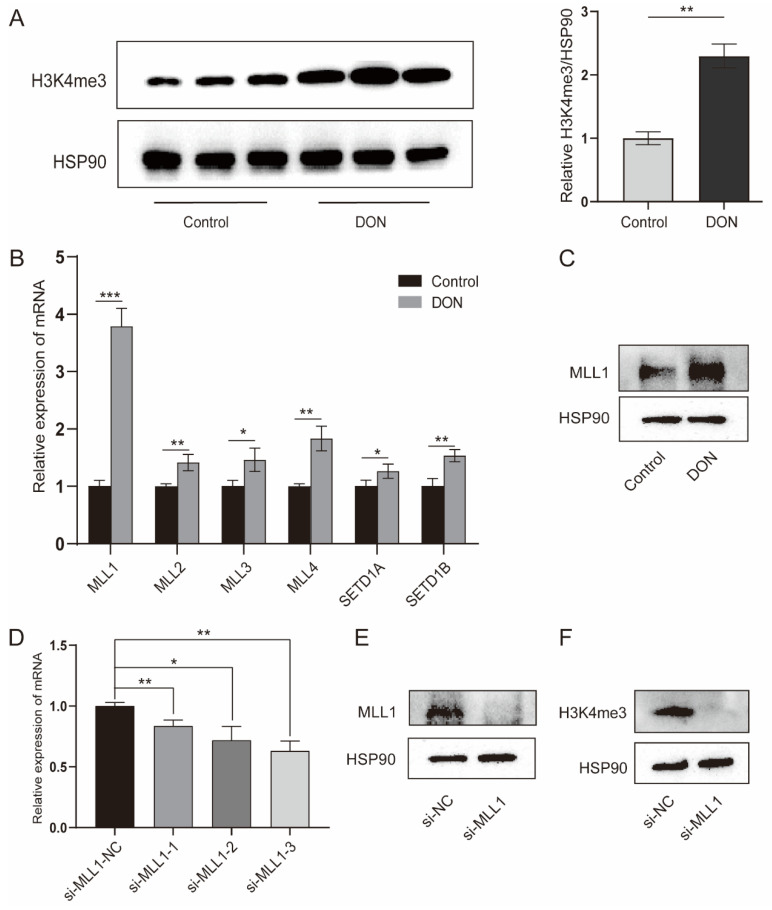
Upon DON exposure, the H3K4me3 enrichment was enhanced and the expression of histone methyltransferases was elevated. (**A**) The protein level of H3K4me3 in IPEC-J2 cells treated with 1 μg/mL DON or not at 48 h was determined by western blot. (**B**,**C**) The mRNA abundances of histone methyltransferases were detected by qPCR and the MLL1 protein level was examined by western blot. (**D**) The IPEC-J2 cells were transfected with *MLL1* small interfering RNA (siRNA) and after 48 h the cells were harvested to check the knockdown efficiency. (**E**,**F**) The protein levels of MLL1 and H3K4me3 were measured after cells were transfected with siRNA for 48 h. Data are presented as mean ± SD, * *p* < 0.05, ** *p* < 0.01, *** *p* < 0.001 (Student’s *t*-test).

The H3K4me3 modification was significantly enriched by 1 μg/mL DON treatment (*p* < 0.01) (Figure 2A). H3K4me3 is a dynamic process that is regulated by histone methyltransferases and demethylases. We further investigated a variety of genes that participate in the H3K4me3, the methyltransferase genes (*MLL1*, *MLL2*, *MLL3*, *MLL4*, *MLL5*, *SETD1A* and *SETD1B*) were increased with 1 μg/mL DON treatment (Figure 2B); especially, *MLL1* was markedly elevated. As shown in Figure 2C, we found that DON increased the MLL1 protein. The IPEC-J2 cells transfected with si-*MLL1*-3 possessed the highest knockdown efficiency (*p* < 0.01) (Figure 2D). The protein level of MLL1 was obviously reduced by treatment with si-*MLL1* (Figure 2E), and H3K4me3 was also subsequently declined (Figure 2F). These above-mentioned results suggest that *MLL1* may participate in the regulation of DON induced IPEC-J2 cells.

### 3.3. MLL1 Knockdown Resulted in Cell Cycle Arrest and Exacerbated the Oxidative Stress

**Figure 3:**
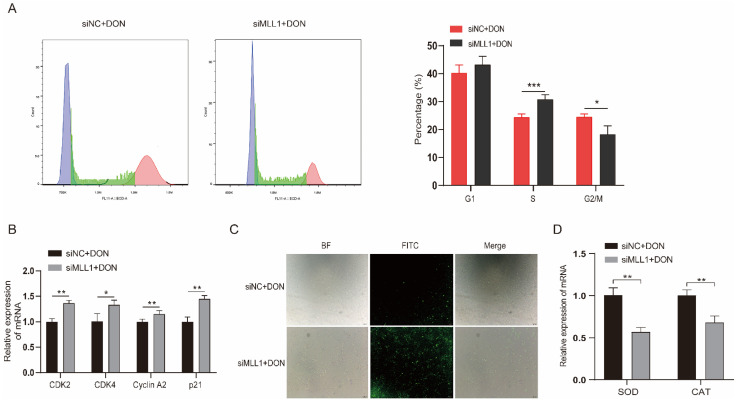
Loss of *MLL1* induced cell cycle arrest and oxidative stress. (**A**) The cell cycle distribution was analyzed by flow cytometry. (**B**) The mRNA levels of cell cycle genes (*CDK2*, *CDK4*, *Cyclin A2* and *p21*) were examined via qPCR. (**C**) Cells were stained with DCHF-DA to detect the reactive oxygen species (ROS) levels. The intensity of FITC (green) shows the level of ROS. Scale bar = 200 µm. (**D**) The mRNA levels of antioxidant genes were determined by qPCR. Data are presented as mean ± SD, * *p* < 0.05, ** *p* < 0.01, *** *p* < 0.001 (Student *t*-test).

Ample reports suggest that DON can arrest the cell cycle and induce ROS in many cell lines [26,27]. The flow cytometry and fluorescent probe were exerted to investigate whether *MLL1* participates in the cell cycle and ROS biological process and plays potential functions. The cell cycle assay showed that the rate of the S phase increased and G2/M phase declined after knockdown of *MLL1* (Figure 3A). The mRNA expression levels of cell cycle related genes (*CDK2*, *CDK4*, *Cyclin A2* and *p21*) were all upregulated (Figure 3B). The ROS levels were elevated after treatment with *MLL1* siRNA (Figure 3C). Furthermore, the expression of antioxidative genes, *SOD* and *CAT*, decreased by si-*MLL1* (Figure 3D). Taken together, the findings indicated that the knockdown of *MLL1* caused cell cycle arrest at the S phase and intensified the ROS.

### 3.4. MLL1 Knockdown Aggravated Cell Apoptosis

**Figure 4:**
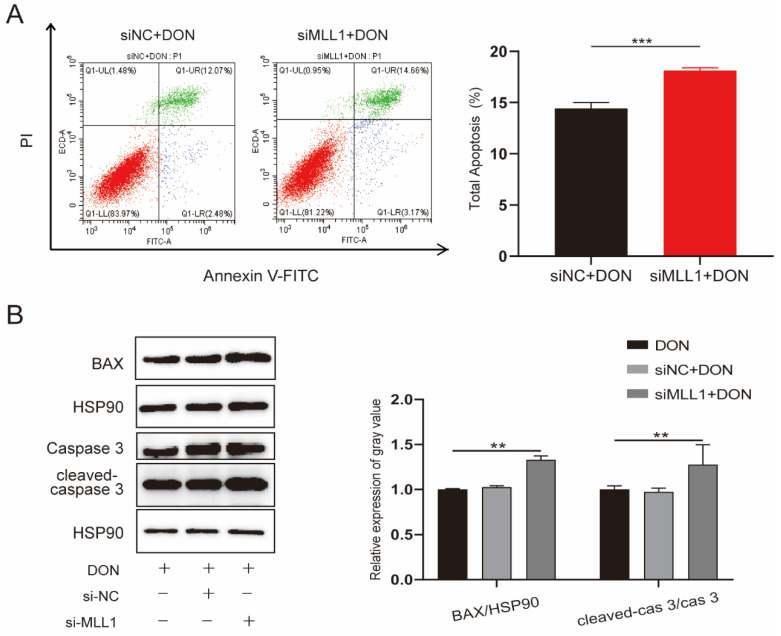
*MLL1* knockdown elevated apoptosis in IPEC-J2 cells exposed to DON. (**A**) Apoptosis of IPEC-J2 cells was detected by flow cytometry. (**B**) Cell apoptosis was determined by protein level of cleaved-caspase-3/caspase 3 and BAX. Data are shown as mean ± SD, ** *p* < 0.01, *** *p* < 0.001 (Student’s *t*-test).

The increasing ROS level was closely associated with apoptosis and was proved by a variety of studies [28,29]. The IPEC-J2 cells were transfected with si-*MLL1*-NC and si-*MLL1* to further test the influence of *MLL1* on apoptosis upon DON exposure. As shown in Figure 4A, the rate of apoptotic cells was elevated by *MLL1* knockdown (*p* < 0.001). Furthermore, the proteins of cleaved-caspase 3/caspase 3 and Bax were upregulated (Figure 4B). Collectively, the results suggested that the loss of *MLL1* indeed aggravated apoptosis induced by DON.

### 3.5. RNA-Sequencing Analysis between si-NC and si-MLL1 Transfected into IPEC-J2 Cells

**Figure 5:**
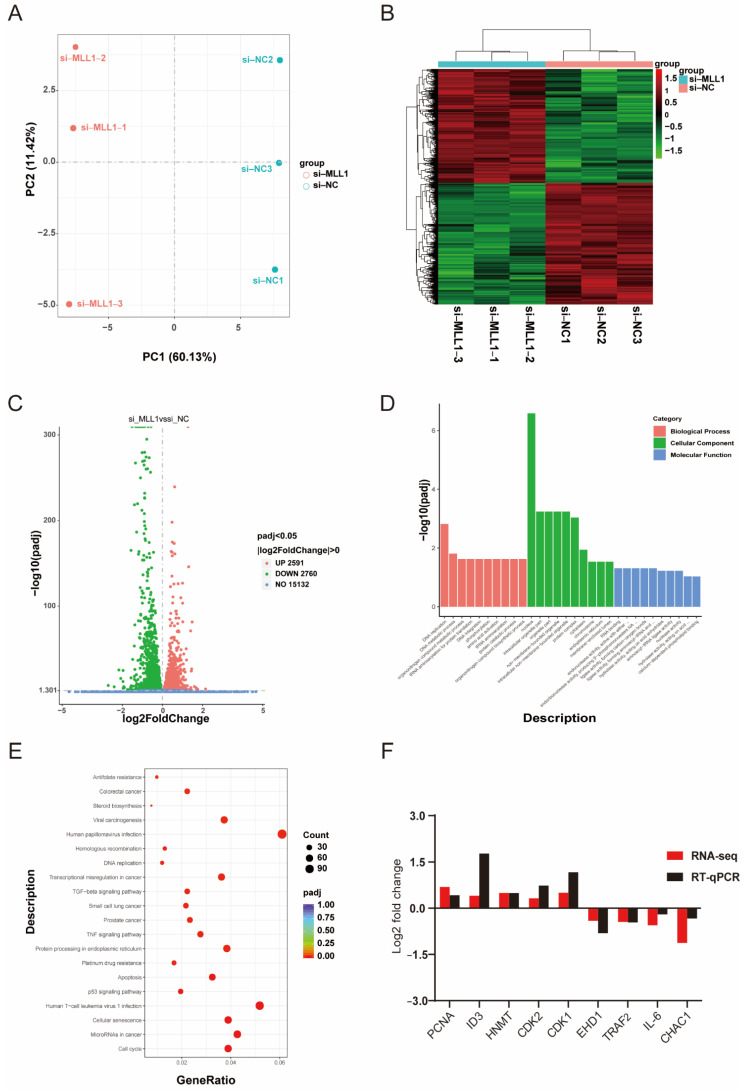
Analysis of RNA-sequencing profile and differentially expressed genes (DEGs). (**A**) The principal component analysis (PCA) between si-*MLL1*-NC and si-*MLL1* groups; PC1 means 60.13% of the variance and PC2 means 11.42% of the variance. (**B**) The cluster heatmap analysis of RNA-seq samples. (**C**) Volcano plot of differential expression genes. Green plots represent downregulated genes and red plots represent upregulated genes. (**D**) Gene Ontology (GO) analysis indicates the DEGs were divided into three parts: biological process, cellular component, and molecular function. (**E**) The bubbles showed the top 20 enriched Kyoto Encyclopedia of Genes and Genomes (KEGG) pathway of the DEGs. The dot size means the quantity of DEGs enrichment. (**F**) RNA-seq data were randomly selected and validated by RT-qPCR. Fold changes are shown as the expression level of genes between si-*MLL1*-NC and si-*MLL1* group.

To identify and figure out the underlying mechanism induced by *MLL1*, we performed RNA sequencing. First, principal component analysis (PCA) indicated an obvious separation of the si-*MLL1*-NC group from si-*MLL1* group (Figure 5A), and the heatmap analysis of sample-to-sample correlation also validated this (Figure 5B). Adjusted *p* < 0.05 and |log2 fold change| > 0 were utilized as criteria to filter differentially expressed genes. As shown in Figure 5C, there are 2591 and 2760 mRNAs upregulated and downregulated, respectively (Figure S1). As well, the differentially expressed genes between two groups were provided in Tables S4 and S5. Gene Ontology (GO) enrichment analysis was divided into three terms, including biological process, cellular component, and molecular function (Figure 5D and Figure S1C,D; Table S6). For all DEGs, Kyoto Encyclopedia of Genes and Genomes (KEGG) enrichment analysis indicated that *MLL1* mediates the cell cycle, apoptosis and TNF signaling pathway (Figure 5E; Tables S7 and S8). To validate the reliability of RNA-sequencing analysis, nine genes were randomly selected and their expression levels were examined by qPCR; we then found the result is consistent with the expression changes detected by RNA-seq (Figure 5F). 

### 3.6. Loss Function of MLL1 Exacerbated MAPKs Activation

**Figure 6:**
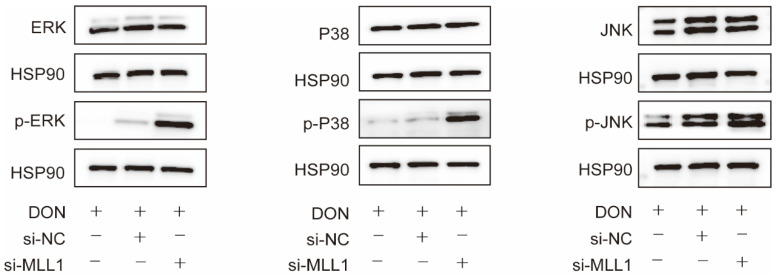
Loss of *MLL1* activated the MAPKs inIPEC-J2 cells. The protein levels of p-ERK, p-p38 and p-JNK were examined by western blot.

Accumulating reports have validated that ROS could trigger the MAPKs signaling pathway and lead to the secretion of pro-inflammatory cytokines which promotes inflammation response [30,31]. Western blot analysis represented that the phosphorylated protein levels of p38, ERK, and JNK markedly increased after the IPEC-J2 cells were transfected with si-*MLL1* compared with si-*MLL1*-NC upon 1 μg/mL DON exposure (Figure 6).

### 3.7. Identification of TNFRSF1A Core Promoter Region and Function of TNFRSF1A

**Figure 7:**
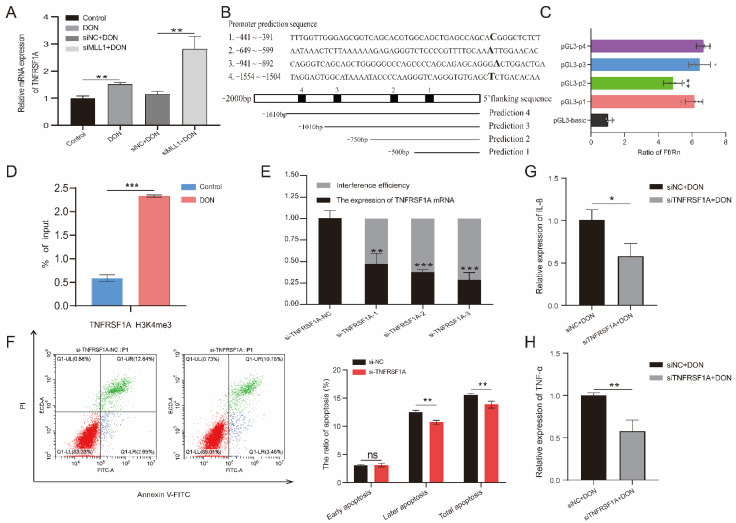
The effects of TNFRSF1A in regulating DON induced cytotoxicity. (**A**) The relative expression of *TNFRSF1A* was determined by qPCR among Control, DON, si-*TNFRSF1A*-NC+DON and si-*TNFRSF1A*+DON group. (**B**) Prediction of *TNFRSF1A* gene promoter region and amplified the corresponding truncated sequences. (**C**) Identified the core promoter region via dual-luciferase reporter. (**D**) The H3K4me3 modification at the *TNFRSF1A* promoter was analyzed by ChIP-qPCR assay after the IPEC-J2 cells was treated with 1 μg/mL DON. The result is shown as % of input. (**E**) The interference efficiency of *TNFRSF1A* mRNA examined by qPCR. (**F**) Flow cytometry analysis the cell apoptotic ratio after knockdown of *TNFRSF1A*. (**G**,**H**) Relative mRNA expression of *IL-8* and *TNF-α* after silenced *TNFRSF1A*. All data are presented as the mean ± SD, * *p* < 0.05, ** *p* < 0.01, *** *p* < 0.001, ns > 0.05 (Student *t*-test).

According to the RNA-seq, we found that TNFRSF1A may be the potential target of *MLL1*. Previous studies indicated that tumor necrosis factor receptor 1 (TNFR1), which is encoded by TNFRSF1A, is a hub to mediate the apoptosis and inflammation response pathway [32]. The qPCR that detected the relative mRNA expression of *TNFRSF1A* was elevated when induced by 1 μg/mL DON, and knockdown of *MLL1* also obviously increased its expression (Figure 7A). According to the prediction of the *TNFRSF1A* core promoter region from the website, we amplified the truncated products and recombined it with the pGL3-basic vector to construct four insertional vectors (Figure 7B and Figure S2). Then we performed a dual-luciferase reporter and identified that the luciferase activity of pGL3-p1 was remarkedly higher than the pGL3-basic (*p* < 0.01) (Figure 7C), which suggested that the core promoter region of *TNFRSF1A* was located at −441~−391. Furthermore, we also examined that H3K4me3 modification was significantly enriched in the *TNFRSF1A* promoter region through a ChIP-qPCR (*p* < 0.001) (Figure 7D and Figure S3). Afterwards, we constructed a small interfering RNA of *TNFRSF1A* and validated the si-*TNFRSF1A*-3 possessing the best interference efficiency (Figure 7E). Silencing of *TNFRSF1A* resulted in the rate of later apoptosis being increased when a flow cytometry assay was used (Figure 7F). Moreover, knockdown of *TNFRSF1A* led to the relative expression of *TNF-α* and *IL-8* being declined upon 1 μg/mL DON exposure (Figure 7G,H).

## 4. Discussion

DON is one of the most prevalent environmental toxins that is produced by fungi, which attracts cytotoxicity and causes immune system dysregulation and is severely harmful to animals and human health [33]. Furthermore, recently accumulating studies uncovered that mycotoxins could elicit different types of cancer [34]. In 2016, the International Agency for Research on Cancer (IARC) and World Health Organization (WHO) urged for action to be taken against widespread mycotoxin contamination in developing countries. DON is regarded by the IARC as a Group 3 carcinogen that is suspected of carcinogenicity in humans and there are no adequate human and animal data. Increasing studies explored the effects of DON exposure to the body; however, the potential regulatory mechanism remains to be clarified. 

**Figure 8:**
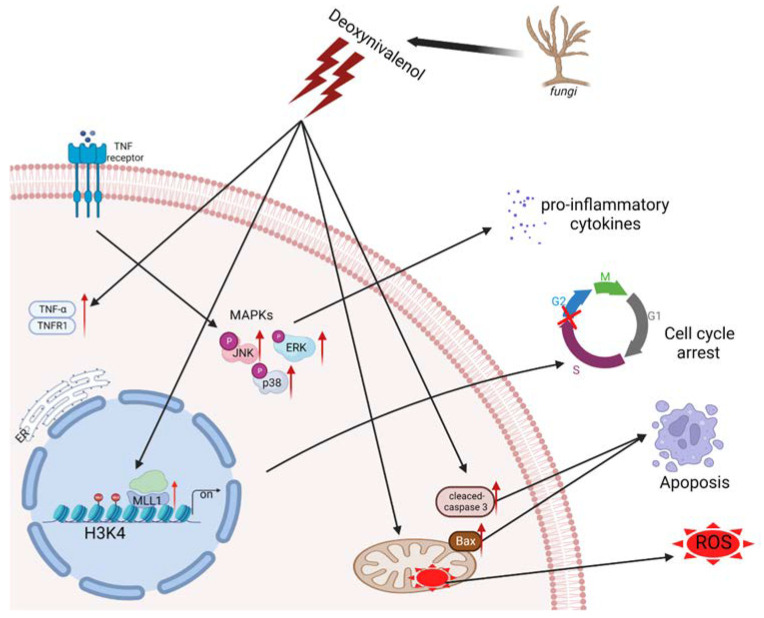
The diagram of regulation mechanism of *MLL1* upon IPEC-J2 cell exposure DON.

Recently, epigenetic modification was reported to be related to the regulation of intracellular oxidative stress response caused by mycotoxin. The protein post-translational modifications have drawn much attention to their ability to regulate gene expression and impact chromatin structure while not directly affecting the sequence of genes [35]. Czakai et al. found that histone acetyltransferases participating in Ochratoxin A (OTA) led to toxicity and carcinogenicity [36]. They reported that OTA caused core histones to be phosphorylated and acetylated, after which the mitotic chromosomes were condensed and the sister chromatid was separated. The histone modification alterations, including the levels of H4K20me3 and H3K9me3 that increased and levels of H4K20me2 and H3K27me3 that decreased, were monitored in the mouse oocytes upon Aflatoxin B1 (AFB1) exposure [37]. In this study, we proved that after IPEC-J2 cells’ exposure to 1 μg/mL DON, H3K4me3 modification was enriched and the gene expression of histone methyltransferases were elevated; in particular, the levels of *MLL1* mRNA expression were dramatically increased. The results suggest that *MLL1* may take part in the regulation of DON-induced cytotoxicity. Consistent with the previous study conducted by Ansari et al., they cultured human H358 cells and treated them with different concentrations of DON (up to 33 µM) for 7.5 h, then observed that the levels of *MLL1* and *Set1* mRNA induced a two- to five-fold upregulation in a concentration-dependent manner, and the protein level of MLL1 was also obviously increased (8.3-fold) [38]. Moreover, they reported that the transcription factor Sp1 plays a crucial role in the regulation of *MLL1* under stress. As shown in Figure 8, we found that *MLL1* plays a crucial role during DON exposure to IPEC-J2 cells. Previous investigations indicated that *MLL1* could promote cell proliferation, self-renewal and skeletal muscle regeneration, and also participate in the biological process, including hematopoiesis and development [39,40]. *MLL1* attracted people’s attention in the study of mixed lineage leukemia. However, the study of its own function in other fields was limited. Besides, *MLL1* was regarded as one of the histone methyltransferases and specifically regulated the methylation on histone 3 lysine 4 [41]. In the present study, a small interfering RNA was further exerted to explore the function of *MLL1*. The silencing of *MLL1* resulted in an attenuation of *MLL1* mRNA and MLL1 protein, and H3K4me3 modification also decreased. Furthermore, cell cycle was arrested after the loss of *MLL1*. A previous study indicated that the knockdown of *MLL1* induced cell cycle arrest at G2/M phase in HeLa cells [42]. Histone methylation modification could regulate gene expression through changing the state and structure of chromatin [43]. In the myogenesis process, Myf5 is considered an important transcription factor. The si-*MLL1* decreased H3K4me3 modification. Therefore, *MLL1* probably directly regulates Myf5 and affects the expression of Cyclin D1, resulting in cell cycle arrests in the G1/S phase [44]. Herein, we also illustrated that the loss function of *MLL1* increased cell apoptosis and ROS levels. A knockdown of *MLL1* declined the expression of p16 ^INK4A^ and induced cellular senescence after cells were treated with ambient air particulate PM with a diameter < 2.5 (PM_2_._5_) [45]. Besides, N-acetylcysteine (NAC), known as an antioxidant, attenuated the ROS increase upon PM_2_._5_ exposure in NHEK and HaCaT cells. The cells were pretreated with NAC followed by PM_2_._5_ treatment; the ROS levels and epigenetic enzymes’ (DNMT, DNMT3B, TET1, EZH2 and MLL1) expression levels returned to normal. In this study, we demonstrated that si-*MLL1* aggravated the apoptosis rate and increased the expression of cleaved-caspase 3 and Bax upon IPEC-J2 cell exposure to 1 μg/mL DON. 

We performed an RNA-sequencing profile between si-*MLL1* and negative control groups to investigate the function of *MLL1*. Adjusted *p* < 0.05 and |log2 fold change| > 0 were used as criteria to screen differentially expressed genes. As shown in Figure 5C, 2591 mRNAs were upregulated and 2760 mRNAs were downregulated. The KEGG enrichment analysis proved that *MLL1* participates in the cell cycle, apoptosis, TNF signaling pathway, and so on. 

The MAPKs include three subfamilies, ERK 1/2, p38 and JNK; its phosphorylated proteins performed the function of regulating inflammation, cell growth, development, and apoptosis [46,47]. The p38/MAPK and ERK 1/2/MAPK are correlated with the inflammation response via mediated proinflammatory cytokines secretion, such as interleukin families and TNF-α [48]. Our study revealed that the silencing of *MLL1* significantly elevated the phosphorylated levels of ERK, p38, and JNK after IPEC-J2 cells were induced by 1 μg/mL DON. These results suggested that oxidative stress probably regulates the MAPKs (ERK, p38 and JNK) signaling pathway to trigger cell apoptosis and cell cycle arrest. 

According to our RNA-seq, TNFRSF1A may be the target to regulate the cytotoxicity and genotoxicity induced by DON. TNFRSF1A is regarded as a transmembrane receptor for TNF-α, and *TNFRSF1A* encodes a protein called TNFR1 which can assist TNF-α to signal into cells [49]. TNFR1 possesses a death domain and is involved in a variety of cellular processes, such as cell apoptosis [50]. Consistent with the previous study, the expression of *TNF-α* and *TNFRSF1A* mRNA was significantly increased when cells upon oxidative stress. In addition, the modification of H3K4me3 was increased in the promoter region of *TNFRSF1A* after IPEC-J2 cells were treated with 1 μg/mL DON. Besides, the silencing of *MLL1* induced an elevation of the mRNA expression of *TNFRSF1A*. Nevertheless, we discovered that a knockdown of *TNFRSF1A* attenuated apoptosis and inhibited the expression of *IL-8* and *TNF-α mRNA*. These evidences suggested that the blocking of TNFR1 attenuated the inflammatory response induced by DON through the TNF-α signaling pathway. 

## 5. Conclusions

In summary, we confirmed that a histone methyltransferase MLL1 and modification of H3K4me3 significantly increased upon DON exposure, and the knockdown of *MLL1* could aggravate ROS levels, cell apoptosis, induce cell cycle arrest and activate the MAPKs pathway. Moreover, we also performed an RNA-seq profile to identify the *MLL1* potential target gene. We provide new insight that *MLL1* probably mediates the H3K4me3 modification of the *TNFRSF1A* promoter region to regulate gene expression and take part in DON-induced cytotoxicity.

## Data Availability

The data presented in this study are available on request from the corresponding author.

## Supplementary Materials

The following supporting information can be downloaded at: https://www.mdpi.com/article/10.3390/antiox11102006/s1, Figure S1: Differentially expressed genes and its function enrichment analysis between *si-MLL1-NC* and *si-MLL1*; Figure S2: Construct the *TNFRSF1A* gene promoter region truncated vector; Figure S3: Evaluation of the condition of Chromatin sonication by agarose gel electrophoresis; Table S1: The siRNA sequences of *MLL1* and *TNFRSF1A*; Table S2: The primers of RT-qPCR; Table S3: Amplification primers of different fragments of pig *TNFRSF1A* gene promotor region & The primers of ChIP-qPCR; Table S4: Differentially Expression Genes of up-regulation (*si-MLL1* vs. *si-MLL1-NC*); Table S5: Differentially Expression Genes of down-regulation (*si-MLL1* vs. *si-MLL1-NC*); Table S6: GO enrichment analysis of DEGs; Table S7: KEGG enrichment analysis of up-regulated DEGs, Table S8: KEGG enrichment analysis of down-regulated DEGs.
