# Supplementary material for: Histone Methyltransferase MLL1 Mediates Oxidative Stress and Apoptosis upon Deoxynivalenol Exposure in the Intestinal Porcine Epithelial Cells
Source: Antioxidants (Basel). 2022 Oct 11;11(10):2006. doi: 10.3390/antiox11102006 (PMC9598511; doi:10.3390/antiox11102006)
Supplement: Supplementary file 1 [file antioxidants-11-02006-s001.zip › antioxidants-1901236-supplementary/Supplementary Materials/Supplementary Figures.pdf]

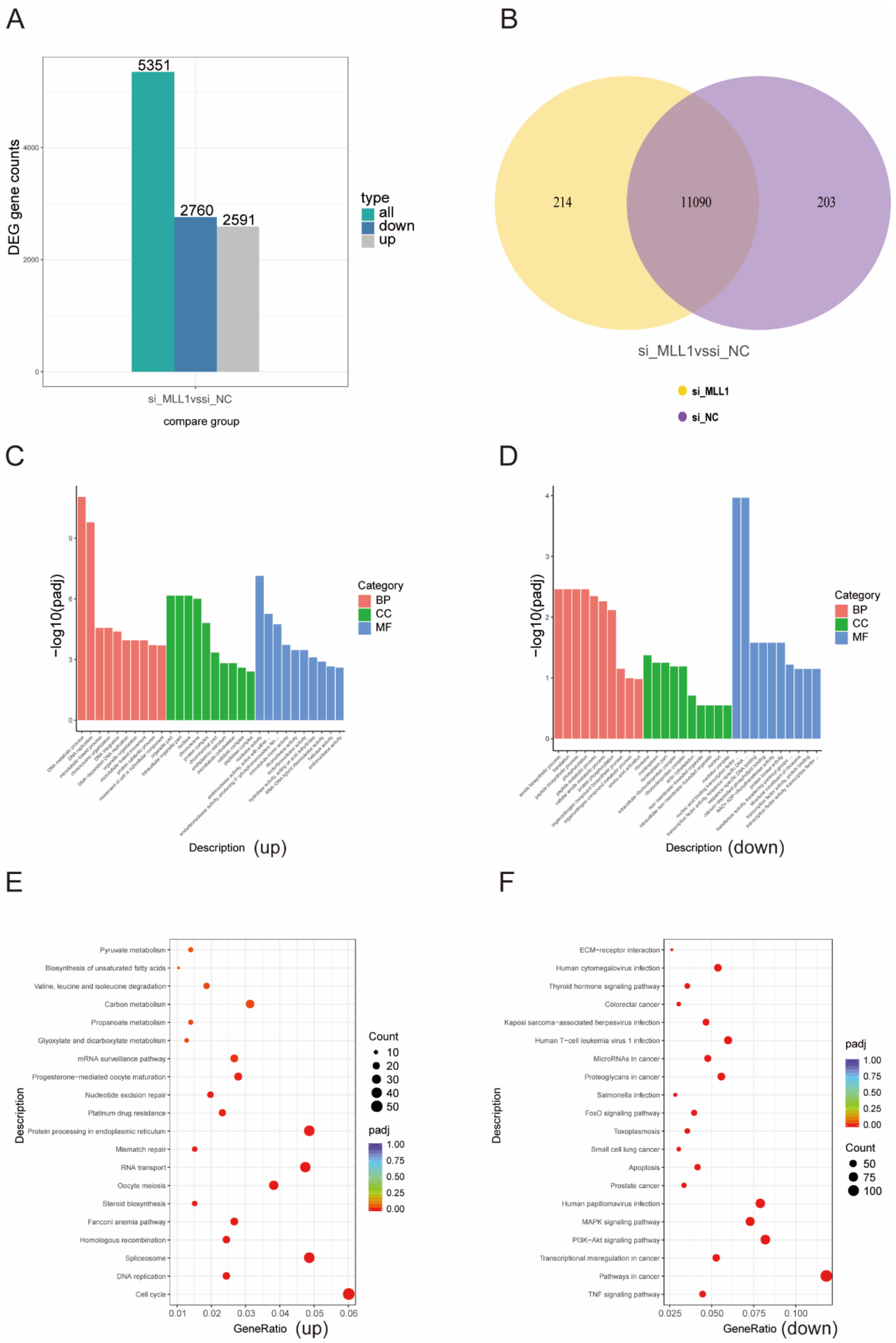

Figure S1. Differentially expressed genes and its function enrichment analysis between si-MLL1-NC and si-MLL1. (**A**) DEGs counts, including 2760 of down-regulated and 2591 of up-regulated. (**B**) Venn diagram of DEGs. (**C, D**) GO enrichment analysis of up-regulated and down-

regulated DEGs. (**E, F**) KEGG enrichment analysis of up-regulated and down-regulated DEGs.

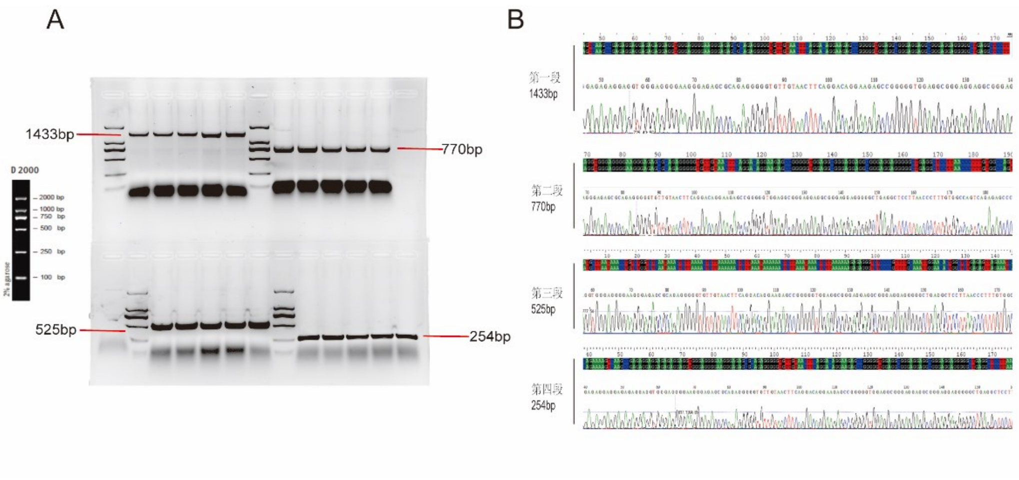

Figure S2. Construct the TNFRSF1A gene promoter region truncated vector. (**A**) PCR amplification of different fragments of *TNFRSF1A* gene promoter. (**B**) PCR sequencing results of the truncated plasmids (pGL3-TNFRSF1A-p1, pGL3-TNFRSF1A-p2, pGL3-TNFRSF1A-p3, pGL3-TNFRSF1A-p4).

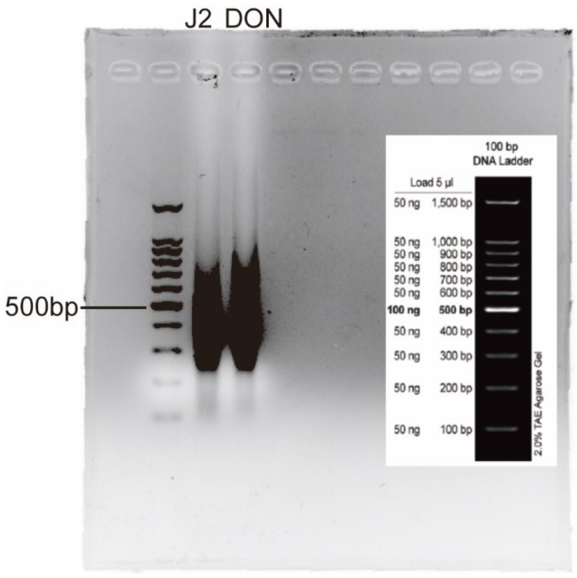

Figure S3. Evaluation of the condition of Chromatin sonication by agarose gel electrophoresis.
